# Functional expression of TRPV6 in human syncytiotrophoblasts from trophoblast stem cells – possible role in maternal-fetal calcium transport

**DOI:** 10.1016/j.jphyss.2026.100099

**Published:** 2026-08-31

**Authors:** Yoshiro Suzuki, Soshi Otani, Shouichi Shibasaki, Keishi Otsu, Katsumi Nakajima

**Affiliations:** a Division of Integrative Physiology, Department of Physiology, Iwate Medical University, 1-1-1 Idaidori, Yahaba-cho, Shiwa-gun, Iwate 028-3694, Japan; b Division of Developmental Biology and Regenerative Medicine, Department of Anatomy, Iwate Medical University, 1-1-1 Idaidori, Yahaba-cho, Shiwa-gun, Iwate 028-3694, Japan

**Keywords:** TRPV4, TRPV6, Placenta, Maternal-fetal calcium transport, Cell fusion

## Abstract

Maternal-fetal calcium (Ca^2 +^) transport through the placenta is crucial for the development of fetal organs including bones. Although we have reported that the transient receptor potential cation channel, subfamily V, member 6 (TRPV6) was responsible for the maternal-fetal Ca^2+^ transport, there has been no direct evidence for its channel activity in placental trophoblasts. By using human trophoblast stem cells, we observed biphasic higher intracellular Ca^2+^ levels compared with undifferentiated cells during the differentiation of syncytiotrophoblasts. Second Ca^2+^ peak, when the syncytiotrophoblasts might mature with transport function, was found to be a Ca^2+^ flux through TRPV6. Unexpectedly we observed the first peak, when trophoblasts fuse to form syncytiotrophoblast. We found that it was derived through TRPV4, and blocking TRPV4 suppressed trophoblast cell fusion. Our results might provide the first evidence for TRPV6 channel activities in syncytiotrophoblasts involved in the maternal-fetal Ca^2+^ transport.

## Introduction

Maternal-fetal calcium (Ca^2+^) transport through the placenta is critical for fetal Ca^2+^ homeostasis to maintain fetal development including brain and heart, in parallel with supplying Ca^2+^ for fetal bone mineralization [1]. We have shown that the transient receptor potential cation channel, subfamily V, member 6 (TRPV6) is involved in this maternal-fetal Ca^2+^ transport through the placenta [2]. Moreover, mutations in the gene encoding TRPV6 have been reported to be associated with transient neonatal hyperparathyroidism with bone abnormalities in humans [3], [4], [5], [6], [7]. However, little is known about the physiological significance and regulation of TRPV6 in the Ca^2+^ transport, since there has been no report showing TRPV6 activity in the placenta.

In the placenta, maternal-fetal nutrient transport including Ca^2+^ is thought to be conducted by syncytiotrophoblasts, that are formed from cytotrophoblast cell fusions. To elucidate functional significance of TRPV6 in the placenta, we investigated endogenous TRPV6 physiologically and molecular biologically in syncytiotrophoblasts that were differentiated from trophoblast stem cells [8].

## Materials and methods

### Culture of human trophoblast stem cells and differentiation into syncytiotrophoblasts

Human trophoblast stem cells (CT27, RCB4936) were obtained from Riken Cell Bank (Japan). Cells were maintained as described in the literature [8]. Briefly, CT27 cells were cultured in a type Ⅳ collagen (Corning) -coated dish with DMEM/F12 medium (Wako) containing 2-mercaptoethanol (0.1 mM, Thermo Fisher), FBS (0.2%, Thermo Fisher), penicillin-streptomycin (0.5%, Gibco), BSA (0.3%, Wako), ITS-X (1%, Wako), ascorbic acid (1.5 µg/ml, Wako), EGF (50 ng/ml, Wako), CHIR99021 (2 µM, Wako), A83–01 (0.5 µM, Wako), SB431542 (1 µM, Wako), VPA (0.8 mM, Wako) and Y27632 (5 µM, Wako) at 37℃ in 5% CO_2_. Medium was replaced every 2 days. Before 100% confluence, cells were passaged to a new plate by dissociating cells with TrypLE (Wako) at 37℃ for 15 min. For the differentiation into syncytiotrophoblasts [8], medium was replaced into DMEM/F12 medium (ST2D medium) containing 2-mercaptoethanol (0.1 mM), penicillin-streptomycin (0.5%), BSA (0.3%), ITS-X (1%), Y27632 (5 µM), forskolin (2 µM, Wako), and KSR (4%, Gibco). ST2D medium was reported to be suitable for 2D culture such as conventional culture plate which was used in this study. For the differentiation into the extravillous trophoblasts (EVT) [8], medium was replaced into DMEM/F12 medium (EVT medium) containing 2-mercaptoethanol (0.1 mM), penicillin-streptomycin (0.5%), BSA (0.3%), ITS-X (1%), NRG1 (100 ng/ml, Peptide Institute, Inc.), A83–01 (7.5 µM), Y27632 (2.5 µM), and KSR (4%). iMatrix511 (0.25%, Wako) was added when suspending the cells in the medium. Medium was replaced every 2 days.

### Quantitative PCR

Total RNA was isolated using Nucleospin RNA kit (Takara), and first strand cDNA was synthesized using PrimeScript RT Master Mix (Takara). Quantitative PCR was performed using KAPA SYBR Fast qPCR kit (Roche) with 10 µL KAPA SYBR FAST, 0.2 µM specific primer sets (SDC1-f: 5’-GATCAAGATGGCTCTGGGGA, SDC1-r: 5’-AGGCTGCATGTCCCTGTG, HLAG-f: 5’-AACCCTCTTCCTGCTGCTC, HLAG-r: 5’-CACTTGCGCTTGGAGATCTG, TRPV6-f: 5’-TGTCACTGCCCAAGGAGAAA, TRPV6-r: 5’-TATGTGTAGCGCTGTTTCCC, GAPDH-f: 5’-CACATCGCTCAGACACCATG, GAPDH-r: 5’-TTGATGACAAGCTTCCCGTT), 0.4 µL ROX Low, and 1 µL of the prepared cDNA. Quantitative PCR analysis was carried out using an Applied Biosystems 7500 Fast Real-Time PCR System. Temperature profile consisted of 40 cycles of denaturation at 95℃ for 2 s, and annealing/elongation at 60℃ for 20 s. To discriminate specific amplification from non-specific ones, a dissociation stage was added at the end of each PCR assay to analyze melting curves. To determine the starting cDNA amount, purified PCR fragments with known concentration were serially diluted and used as standards.

### Immunocytochemistry

Cells were fixed with 4% paraformaldehyde/PBS for 30 min at room temperature, rinsed 3 times with PBS, then treated with a blocking solution (0.4% saponin, 1% BSA, 2% normal goat serum in PBS) overnight at 4℃. Fixed cells were then incubated with rabbit anti-E-cadherin (1/500, Cell Signaling Technology #3195), SDC1 (1/500, Cell Signaling Technology #96489), HLA-G (1/500, Cell Signaling Technology #79769), or TRPV6 (1/500, Alomone #ACC-036) antibody or without primary antibody with the blocking solution overnight at 4℃. After washing 3 times with PBS, cells were treated with anti-rabbit IgG-Alexa488 (1/200. Life Technologies Inc #A11034) and Hoechst33342 (Dojindo) with the blocking solution for 2 h at room temperature. Cells were washed 3 times with PBS, then mounted with Fluoromount (Diagnostic BioSystems, USA). Fluorescent signals were visualized by BZ-9000 all-in-one microscope (KYENCE, Japan). Percentage of positive area (%Area) was analyzed using ImageJ software.

### Intracellular Ca^2+^ imaging

Intracellular Ca^2+^ imaging was carried out as described previously [7]. Cells were incubated with Fura-2-AM (5 µM) (Dojindo) at 37℃ for 1 h. Intracellular Ca^2+^ concentration of human trophoblasts was analyzed in a standard extracellular solution containing 143 mM NaCl, 5 mM KCl, 2 mM CaCl_2_, 2 mM Mg Cl_2_, 10 mM glucose, and 5 mM HEPES (pH 7.4). To inhibit TRPV6 channel activity, cis-22a (5 µM, Sigma-Aldrich #SML3112) [9], [10] was added at day 0 for the first peak or day 4 for the second peak. To block TRPV4, GSK2193874 (500 nM, Selleck #S8367) was added at day 0 for the first peak or day 4 for the second peak. Ratio-metric imaging was performed at 340 nm and 380 nm, and emitted light was read at 510 nm with a CCD camera (Cooke Pixelfly 12 bit CCD camera). This camera was operated by InCyt IM2 system (Intracellular Imaging Inc. USA), containing TS-100 microscope (Nikon), LAMBDA 10-B optical filter changer (Sutter Instrument) with 340 and 380 fluorescent filters. Finally, the ratio of 340/380 was acquired with InCyt2 software (Intracellular Imaging Inc.), and the average ratio of 30 s was used.

### Fusion index

To investigate the differentiation from stem cells into syncytiotrophoblasts, cell fusion was analyzed as described in the literature [11]. Cells were treated with Di-ANEPPS (2 µM, Potentiometric Probes) with Hoechst33342 (Dojindo) at 37℃ for 15 min with 5% CO_2_. Images were acquired using a Nikon A1R confocal microscope. Cell fusion was quantified by calculating fusion index: number of fused nuclei/total nuclear number. Cells with two nuclei were not counted because of possible cell division.

### Statistical analysis

Statistical analysis was performed using SigmaPlot 12.0. Holm-Sidak test was used for quantitative PCR. Mann-Whytney rank-sum test was used for the intracellular Ca^2+^ imaging because we were not sure that the data follow normal distribution. Data were represented as mean ± standard error of the mean (SEM). Symbols * or ** represent significant significance of *p* < 0.05 or *p* < 0.001, respectively.

## Results

**Fig. 1:**
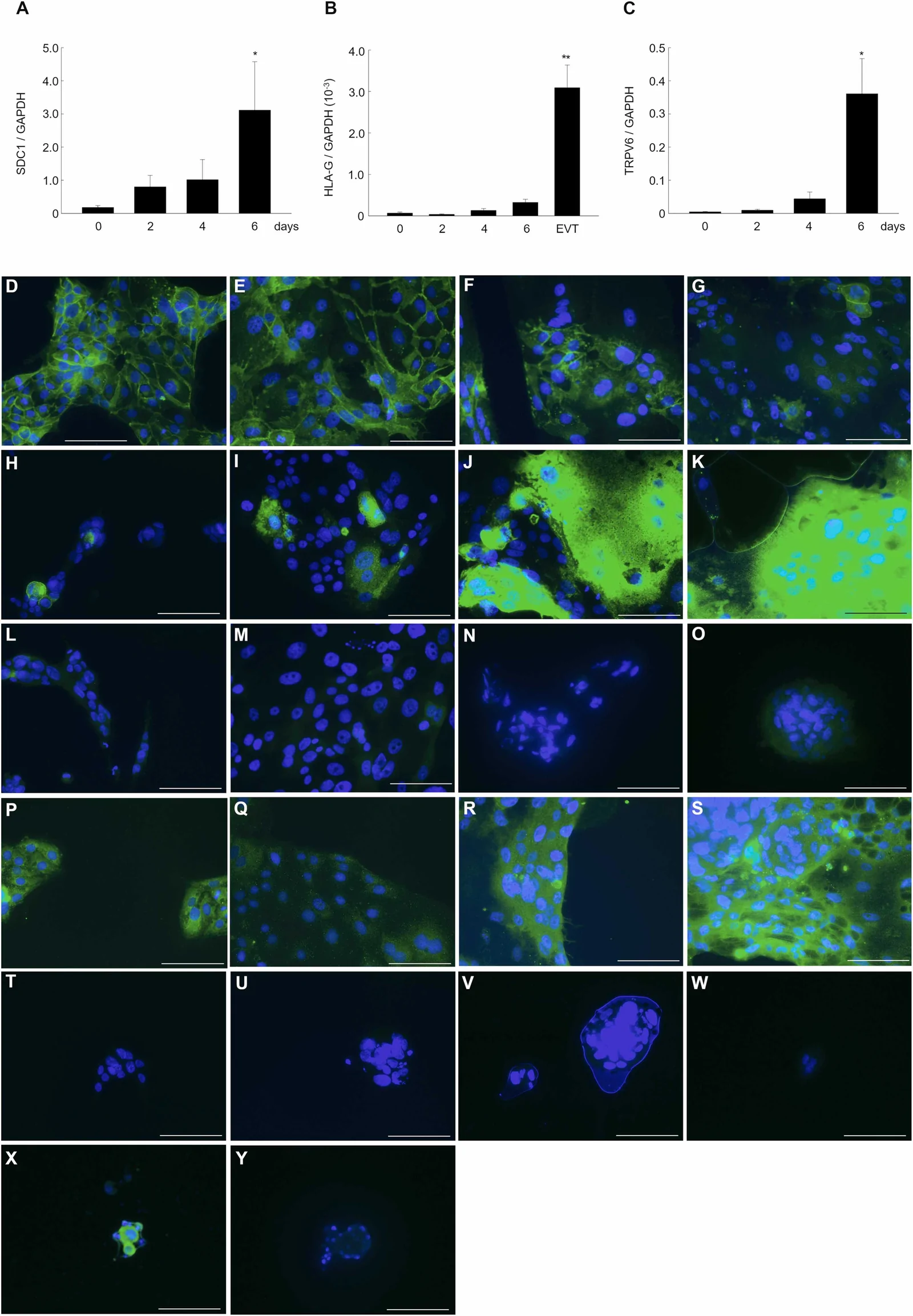
Human trophoblast stem cells differentiated into syncytiotrophoblasts. A-C: Quantitative PCR of human trophoblasts with a marker for syncytiotrophoblasts (SDC1, syndecan 1) (A), a marker for extravillous trophoblasts (HLA-G, human leukocyte antigen G) (B), or TRPV6 (C). *p < 0.05, **p < 0.001, Holm-Sidak test. D-Y: Immunocytochemistry with E-cadherin (D-G), SDC1 (H-K), HLA-G (L-O), TRPV6 (P-S), or cells without primary antibody (T-W) under ST2D medium for 0 days (D,H,L,P,T), 2 days (E,I,M,Q,U), 4 days (F,J,N,R,V), or 6 days (G,K,O,S,W). X-Y: Immunocytochemistry with (X) or without (Y) HLA-G antibody under EVT medium for 6 days. Green signals indicate specific proteins. Blue signal shows nuclei. Bars = 100 µm.

To confirm that human trophoblast stem cells [8] mainly differentiated into syncytiotrophoblasts with ST2D differentiation medium, we performed quantitative PCR and immunocytochemistry of CT27 cells cultured with ST2D medium from day 0 (Fig. 1). A syncytiotrophoblast marker SDC1 (syndecan 1) was observed in mRNA level from day 2, and higher in day 6 (Fig. 1A). SDC1 proteins were also observed from day 2 (Fig. 1I), and its expression levels were higher on day 4 and 6 (Fig. 1J and K). As a consequence of cell fusions, many nuclei were observed in the same cell (Fig. 1K), which was a hallmark of the syncytiotrophoblasts. It was reported that the trophoblast stem cells could differentiate into the extravillous trophoblasts with different culture conditions. An extravillous trophoblast marker HLA-G (human leukocyte antigen G) was expressed very slightly in mRNA level (Fig. 1B) but not expressed in protein level (Fig. 1L-O) when cultured with ST2D differentiation medium. On the other hand, HLA-G mRNA level was much higher when cultured with extravillous trophoblast (EVT) medium (*p* < 0.001, n = 4, Holm-Sidak test, Fig. 1B). Moreover, HLA-G proteins were observed only with EVT medium (Fig. 1X). These strongly suggested that CT27 cells differentiated into EVT only with EVT medium. E-cadherin, a marker for stem cells, decreased during this time course (Fig. 1D-G), suggesting there were almost no stem cells on day 6. Taken together, these results suggested that CT27 cells with ST2D medium differentiated into syncytiotrophoblasts as previously reported [8]. Using the same culture condition, we analyzed the endogenous TRPV6 expressed in these cells. Quantitative PCR indicated that its mRNA expression was higher in day 6 (*p* = 0.001, n = 4, Holm Sidak test, Fig. 1C). TRPV6 proteins were observed predominantly on day 6 (Fig. 1S), although %Area analysis with lower magnification (x20) did not show statistically significant difference (*p* = 0.112, n = 2–4 specimens, one way ANOVA, Suppl. Fig.1). These results suggested that TRPV6 proteins were distributed most likely on matured syncytiotrophoblasts.

**Fig. 2:**
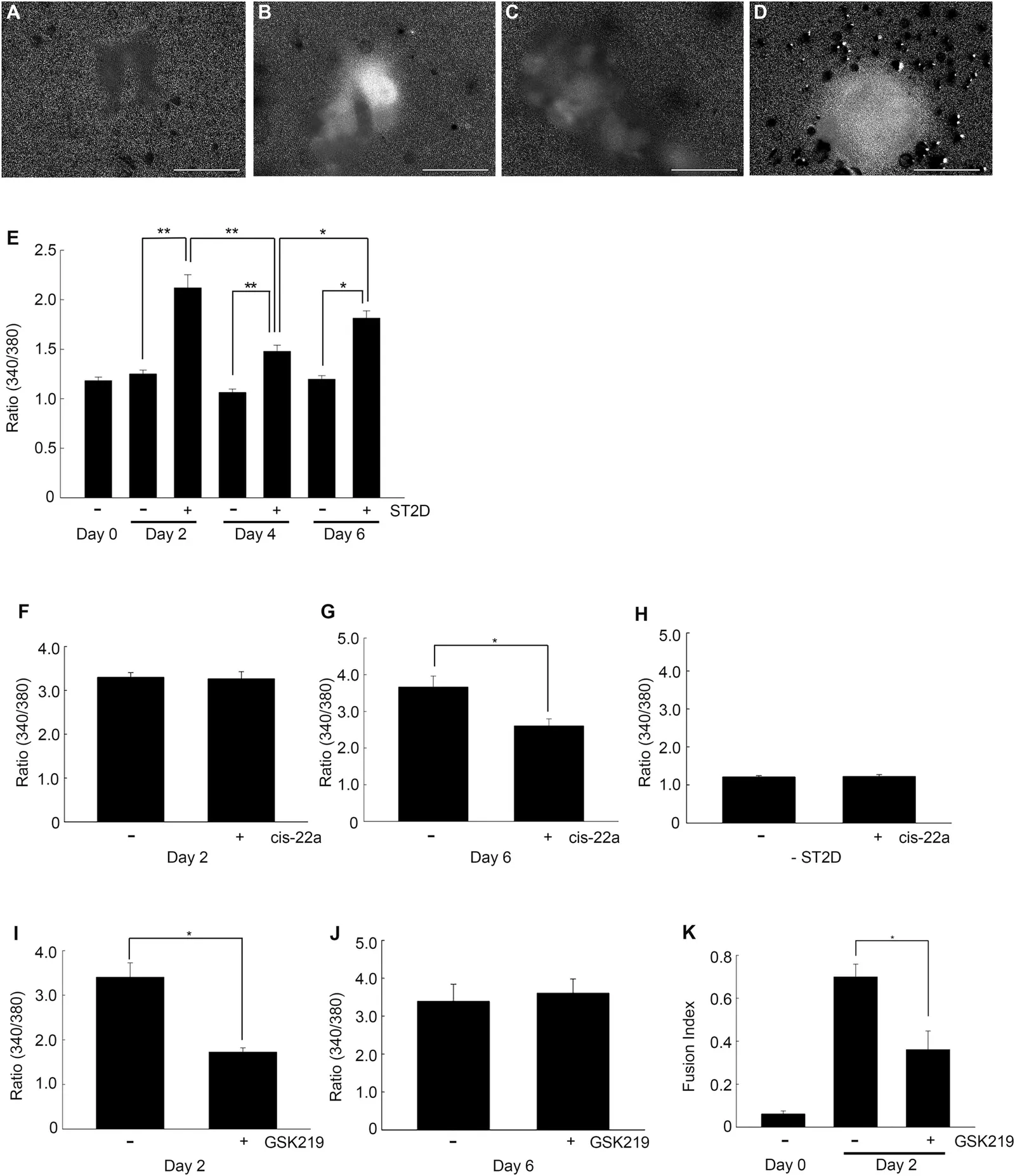
Intracellular calcium levels during the differentiation and maturation of syncytiotrophoblasts. A-D: Representative ratio images of trophoblasts under ST2D medium for 0 days (A), 2 days (B), 4 days (C) or 6 days (D). White signals indicate cells with higher intracellular Ca^2+^ concentration. Bars = 100 µm. E: Intracellular Ca^2+^ levels in trophoblasts with or without ST2D medium. F-H: [Ca^2+^]_i_ levels with or without TRPV6 inhibitor cis-22a (5 µM). I-J: [Ca^2+^]_i_ levels with or without TRPV4 inhibitor GSK2193874 (500 nM). *p < 0.05, **p < 0.001, Mann-Whytney test. K: Fusion index. Cell fusion was quantified by calculating fusion index: number of fused nuclei/total nuclear number. *p < 0.05 (n = 10 images), Mann-Whytney test.

To elucidate the functional significance of the endogenous TRPV6 proteins, we decided to observe the intracellular Ca^2+^ levels ([Ca^2+^]_i_), since we could see a higher [Ca^2+^]_i_ level under an extracellular solution with 2 mM Ca^2+^ when TRPV6 proteins were exogenously expressed in HEK293 cells compared with the non-expression condition [7]. By using intracellular Ca^2+^ imaging, we observed significantly higher [Ca^2+^]_i_ levels only with ST2D differentiation medium. There were significant differences between ST2D(+) and (-) at day 2 (*p* < 0.001, Mann-Whytney test), day 4 (*p* < 0.001, Mann-Whytney test), and day 6 (*p* = 0.004, Mann-Whytney test). However, unexpectedly it was biphasic, peaking on day 2 and day 6 (*p* < 0.001, n = 20–46, Mann-Whytney test, Fig. 2A-E). Then we hypothesized that the second peak on day 6 was derived through TRPV6. Indeed, a specific TRPV6 inhibitor cis-22a [9], [10] significantly suppressed the second peak (day 6, *p* = 0.015, n = 15–21, Mann-Whytney test, Fig. 2G) but not the first peak (day 2, *p* = 0.949, n = 21–26, Mann-Whytney test, Fig. 2F). Cis-22a did not influence the intracellular Ca^2+^ level of undifferentiated trophoblasts without ST2D medium (-ST2D, *p* = 0.891, n = 21–29, Mann-Whytney test, Fig. 2H). These results supported the idea that the Ca^2+^ at the second peak was derived from Ca^2+^ influx through TRPV6.

Regarding the first peak, immunocytochemistry data suggested that it was from ongoing trophoblast cell fusion (Fig. 1E,I,M,Q,U). Since TRPV4 was reported to be involved in this process [11], we blocked TRPV4. Our data indicated that GSK2193874, a specific blocker of TRPV4, significantly suppressed the first peak (*p* = 0.002, n = 8–16, Mann-Whytney test, Fig. 2I) but not the second peak (*p* = 0.341, n = 12–13, Mann-Whytney test, Fig. 2J). Moreover, GSK2193874 suppressed cell fusion itself (*p* = 0.006, n = 10 specimens, Mann-Whytney test, Fig. 2K), suggesting that higher [Ca^2+^]_i_ level in the first peak was derived from Ca^2+^ influx through TRPV4, which was essential for the cell fusion when syncytiotrophoblasts were formed.

## Discussion

Maternal-fetal nutrient transport including Ca^2+^ is important for normal fetal growth. However, little is known about the molecular mechanism because of the limitation of experimental tools. We have reported that TRPV6 plays a role in the maternal-fetal Ca^2+^ transport [2], [3], [4], [5], but there has been no report showing its channel activity in the placenta, except for the ruthenium-red sensitive, TRP-like Ca^2+^ flux in the primary culture of trophoblasts from human term placenta [12], [13]. Here we showed, by using human trophoblast stem cells, a higher intracellular Ca^2+^ level resulting from TRPV6 expression in syncytiotrophoblasts. We concluded that TRPV6 plays a role in maternal-fetal Ca^2+^ transport in the syncytiotrophoblasts. Using this method, molecular mechanism of transient neonatal hyperparathyroidism might be expected to be uncovered by analyzing the TRPV6 disease variants.

Our method, however, had a limitation. Most cells were detached from the culture plate several days after differentiation into syncytiotrophoblasts. To improve this situation, 3D culture might be used [8]. However, it was not very suitable for the intracellular Ca^2+^ imaging because it might be difficult in focusing precisely. Alternative methods using a co-culture with endothelial cells have been reported [14], which might be better tool for investigating the unidirectional nutrient transport including Ca^2+^ in future works.

Unexpectedly, we observed the earlier [Ca^2+^]_i_ peak (first peak) during differentiation process. We showed that, at least in part, it was derived from TRPV4, which has been previously reported to be essential for trophoblast cell fusion [11]. For cell fusion, [Ca^2+^]_i_ increase is necessary because these cells need to activate the lipid scramblase (TMEM16F) for the presentation of phosphatidylserine into cell surface, where the cell fusion starts. In this study we could confirm the higher [Ca^2+^]_i_ level through TRPV4 in the differentiating trophoblasts. This is quite important because now we can start to investigate how TRPV4, an environmental osmotic, temperature, mechanical stress as well as chemical sensor, is activated prior to cell fusion by using this powerful and reproducible procedure. Future works may uncover the entire mechanism of the cell fusion, including trophoblasts, myocytes, melanocytes, osteoclasts, fertilization, as well as the virus-cell membrane fusion such as HIV infection.

## CRediT authorship contribution statement

**Katsumi Nakajima:** Writing – original draft, Supervision, Conceptualization. **Keishi Otsu:** Writing – original draft, Supervision, Conceptualization. **Shouichi Shibasaki:** Investigation, Formal analysis, Conceptualization. **Soshi Otani:** Investigation, Formal analysis, Conceptualization. **Suzuki Yoshiro:** Writing – original draft, Project administration, Methodology, Investigation, Funding acquisition, Formal analysis, Data curation, Conceptualization.

## Funding

This work was supported by JSPS KAKENHI Grant (JP22K06848 and JP25K10158) and KEIRYOKAI grant for YS.

## Declaration of Competing Interest

The authors declare that they have no known competing financial interests or personal relationships that could have appeared to influence the work reported in this paper.

## Data Availability

The scientific data is available on reasonable requests after approval.

## Footnotes

Appendix A Supplementary data associated with this article can be found in the online version at doi:10.1016/j.jphyss.2026.100099.
